# Supplementary material for: DNA Methylation-Based Estimates of Circulating Leukocyte Composition for Predicting Colorectal Cancer Survival: A Prospective Cohort Study
Source: Cancers (Basel). 2021 Jun 12;13(12):2948. doi: 10.3390/cancers13122948 (PMC8231262; doi:10.3390/cancers13122948)
Supplement: Supplementary file 1 [file cancers-13-02948-s001.zip › cancers-1237381-supplementary.pdf]

## Supplementary online content

**Table S1.** Baseline characteristics of the patient cohort according to tumor stages

**Table S2.** Median and interquartile range of leukocyte proportion by characteristics of the study population at baseline.

**Table S3.** Associations of leukocyte composition with all-cause and colorectal cancer (CRC)-specific mortality in total study population (sensitivity analyses).

**Table S4.** Associations of leukocyte composition with all-cause and colorectal cancer (CRC)-specific mortality among patients whose blood sample was taken prior to surgery, more than 1 month after surgery but not receiving chemo- or radiotherapy or more than 5 months after the first chemo- or radiotherapy.

**Figure S1.** Leukocytes proportion according to the time of blood collection relative to treatments.

**Figure S2**. Correlations of leukocyte subtype proportions. ρ: Pearson correlation coefficient.

**Figure S3.** Survival curves for CD4+ T cell proportion by the timing of blood sampling.

**Figure S4.** Adjusted restricted cubic splines for CRC patients across quartiles of CD4+ T cells proportion by therapies.

**Table S1.** Baseline characteristics of the patient cohort according to tumor stages

| Baseline Characteristics | Overall *n* (%) | Stage I *n* (%) | Stage II *n* (%) | Stage III *n* (%) | Stage IV *n* (%) | $P^{\#}$ |
|---|---|---|---|---|---|---|
| **Sex** | | | | | | |
| Women | 910 (41.2) | 151 (37.8) | 315 (41.5) | 321 (44.2) | 118 (37.2) | 0.209 |
| Men | 1296 (58.8) | 249 (62.3) | 444 (58.5) | 405 (55.8) | 191 (61.8) | |
| **Age at diagnosis** | | | | | | |
| 33 -< 65 years | 717 (32.5) | 122 (30.5) | 215 (28.3) | 260 (35.8) | 116 (37.5) | 0.015 |
| 65 -< 75 years | 776 (35.2) | 146 (36.5) | 269 (35.4) | 243 (33.5) | 113 (36.6) | |
| 75 -< 96 years | 713 (32.3) | 132 (33.0) | 275 (36.2) | 223 (30.7) | 80 (25.9) | |
| **BMI at diagnosis*** | | | | | | |
| < 25 $kg/m^2$ | 834 (38.0) | 127 (31.9) | 283 (37.5) | 277 (38.2) | 141 (45.9) | 0.029 |
| 25-30 $kg/m^2$ | 932 (42.4) | 180 (45.2) | 322 (42.7) | 308 (42.5) | 118 (38.4) | |
| > 30 $kg/m^2$ | 430 (19.6) | 91 (22.9) | 150 (19.9) | 140 (19.3) | 48 (15.6) | |
| **Smoking status*** | | | | | | |
| Never | 907 (41.1) | 145 (36.3) | 313 (41.3) | 319 (44.0) | 125 (40.5) | < 0.001 |
| Former | 947 (43.0) | 203 (50.8) | 313 (41.3) | 311 (42.9) | 118 (38.2) | |
| Current | 350 (15.9) | 52 (13.0) | 132 (17.4) | 95 (13.1) | 66 (21.4) | |
| **Charlson comorbidity index** | | | | | | |
| 0 (no comorbidity) | 1281 (58.1) | 233 (58.3) | 402 (53.0) | 439 (60.5) | 200 (64.7) | 0.026 |
| 1 (mild comorbidity) | 479 (21.7) | 80 (20.0) | 182 (24.0) | 155 (21.4) | 59 (19.1) | |
| 2+ (moderate comorbidity) | 446 (20.2) | 87 (21.8) | 175 (23.1) | 132 (18.2) | 50 (16.2) | |
| **Tumor sub-site*** | | | | | | |
| Distal colon † | 738 (33.5) | 123 (30.8) | 277 (36.5) | 223 (30.8) | 114 (37.0) | < 0.001 |
| Proximal colon ‡ | 796 (36.1) | 127 (31.8) | 314 (41.4) | 254 (35.0) | 101 (32.8) | |
| Rectum | 669 (30.4) | 150 (37.5) | 167 (22.0) | 248 (34.2) | 93 (30.2) | |

Note: * 12 missing values for tumor stage, 10 missing values for body mass index, 2 missing values for smoking status and 3 missing values for tumor subsite. Complete case analysis was applied when adjusting for the variables with missing values. † The distal colon includes the descending colon and the sigmoid colon. ‡ The proximal colon includes the cecum, the ascending colon, the right flexure, and the transverse colon and the left flexure. # *P* values were derived from Chi square test of difference in distributions of categorical variables between stages.

**Table S2.** Median and interquartile range of leukocyte proportion by characteristics of the study population at baseline[#]

| Baseline Characteristics | CD4+ T cell | CD8+ T cell | B cell | NK cell | Monocyte | Neutrophil |
|---|---|---|---|---|---|---|
| **Sex** | | | | | | |
| Women | **0.13 (0.09, 0.18)** | **0.07 (0.04, 0.10)** | **0.04 (0.03, 0.05)** | **0.05 (0.04, 0.08)** | **0.07 (0.06, 0.09)** | 0.64 (0.54, 0.73) |
| Men | **0.11 (0.07, 0.16)** | **0.06 (0.04, 0.10)** | **0.03 (0.02, 0.04)** | **0.06 (0.04, 0.08)** | **0.08 (0.06, 0.10)** | 0.65 (0.55, 0.74) |
| **Age at diagnosis** | | | | | | |
| 33 -< 65 year | **0.13 (0.09, 0.18)** | **0.07 (0.04, 0.10)** | **0.04 (0.03, 0.05)** | **0.06 (0.04, 0.08)** | **0.07 (0.06, 0.09)** | 0.64 (0.54, 0.73) |
| 65 -< 75 year | **0.12 (0.08, 0.17)** | **0.06 (0.04, 0.10)** | **0.04 (0.02, 0.05)** | **0.06 (0.04, 0.08)** | **0.07 (0.06, 0.09)** | 0.64 (0.54, 0.73) |
| 75 -< 96 year | **0.11 (0.07, 0.15)** | **0.06 (0.04, 0.10)** | **0.03 (0.02, 0.04)** | **0.06 (0.04, 0.08)** | **0.08 (0.06, 0.10)** | 0.65 (0.56, 0.74) |
| **Tumor stage** | | | | | | |
| I | **0.13 (0.09, 0.17)** | **0.07 (0.04, 0.10)** | **0.04 (0.03, 0.05)** | **0.06 (0.04, 0.10)** | **0.07 (0.06, 0.09)** | **0.62 (0.54, 0.71)** |
| II | **0.12 (0.08, 0.17)** | **0.06 (0.04, 0.10)** | **0.04 (0.03, 0.05)** | **0.06 (0.04, 0.08)** | **0.07 (0.06, 0.09)** | **0.64 (0.54, 0.73)** |
| III | **0.12 (0.07, 0.17)** | **0.06 (0.04, 0.10)** | **0.04 (0.02, 0.05)** | **0.06 (0.04, 0.08)** | **0.08 (0.06, 0.10)** | **0.64 (0.55, 0.74)** |
| IV | **0.11 (0.07, 0.15)** | **0.05 (0.03, 0.09)** | **0.03 (0.02, 0.04)** | **0.05 (0.04, 0.07)** | **0.07 (0.06, 0.09)** | **0.68 (0.56, 0.77)** |
| **Tumor subsite** | | | | | | |
| Distal colon † | **0.13 (0.09, 0.18)** | **0.07 (0.04, 0.10)** | **0.04 (0.03, 0.05)** | **0.06 (0.04, 0.08)** | 0.07 (0.06, 0.09) | **0.62 (0.53, 0.72)** |
| Proximal colon ‡ | **0.13 (0.09, 0.17)** | **0.07 (0.04, 0.11)** | **0.04 (0.03, 0.05)** | **0.06 (0.04, 0.08)** | 0.08 (0.06, 0.09) | **0.63 (0.53, 0.71)** |
| Rectum | **0.10 (0.06, 0.14)** | **0.05 (0.03, 0.09)** | **0.04 (0.02, 0.04)** | **0.05 (0.04, 0.08)** | 0.07 (0.06, 0.09) | **0.69 (0.60, 0.77)** |
| **Body mass index** | | | | | | |
| < 25 $kg/m^2$ | 0.12 (0.07, 0.17) | 0.06 (0.04, 0.09) | **0.04 (0.02, 0.05)** | 0.06 (0.04, 0.08) | 0.08 (0.06, 0.10) | 0.65 (0.55, 0.75) |
| 25-30 $kg/m^2$ | 0.12 (0.08, 0.17) | 0.07 (0.04, 0.10) | **0.03 (0.02, 0.05)** | 0.06 (0.04, 0.08) | 0.07 (0.06, 0.09) | 0.64 (0.54, 0.73) |
| > 30 $kg/m^2$ | 0.12 (0.08, 0.17) | 0.07 (0.04, 0.10) | **0.04 (0.03, 0.05)** | 0.06 (0.04, 0.08) | 0.07 (0.06, 0.09) | 0.64 (0.54, 0.73) |
| **Smoking status** | | | | | | |
| Never | 0.12 (0.08, 0.17) | 0.06 (0.04, 0.10) | **0.04 (0.03, 0.05)** | **0.06 (0.04, 0.08)** | **0.07 (0.06, 0.09)** | 0.65 (0.55, 0.74) |
| Former | 0.12 (0.08, 0.17) | 0.06 (0.04, 0.10) | **0.03 (0.02, 0.05)** | **0.06 (0.04, 0.09)** | **0.08 (0.06, 0.10)** | 0.64 (0.54, 0.73) |
| Current | 0.12 (0.08, 0.18) | 0.06 (0.04, 0.09) | **0.03 (0.02, 0.05)** | **0.05 (0.04, 0.07)** | **0.07 (0.06, 0.09)** | 0.65 (0.55, 0.74) |
| **Charlson Comorbidity Index** | | | | | | |
| 0 (no comorbidity) | **0.13 (0.08, 0.17)** | **0.07 (0.04, 0.10)** | **0.04 (0.03, 0.05)** | 0.06 (0.04, 0.08) | 0.07 (0.06, 0.09) | **0.63 (0.54, 0.72)** |
| 1 (mild comorbidity) | **0.12 (0.07, 0.16)** | **0.06 (0.04, 0.10)** | **0.04 (0.02, 0.05)** | 0.06 (0.04, 0.08) | 0.07 (0.06, 0.09) | **0.66 (0.56, 0.74)** |
| 2+ (moderate comorbidity) | **0.10 (0.07, 0.15)** | **0.06 (0.03, 0.09)** | **0.03 (0.02, 0.04)** | 0.06 (0.04, 0.08) | 0.07 (0.06, 0.09) | **0.67 (0.56, 0.76)** |

Note: # Wilcoxon-Mann-Whitney test was performed for comparing medians across groups of sex and tumor subsite. Jonckheere-Terpstra test was performed to determine the significance of the trend of ordinal variables. * 12 missing values for tumor stage, 10 missing values for body mass index, 2 missing values for smoking status and 3 missing values for tumor subsite. Complete case analysis was applied when adjusting for the variables with missing values. † The distal colon includes the descending colon and the sigmoid colon. ‡ The proximal colon includes the cecum, the ascending colon, the right flexure, and the transverse colon and the left flexure. Numbers printed in bold: Statistically significant difference or trend was observed ($P < 0.05$).

**Table S3.** Associations of leukocyte composition with all-cause and colorectal cancer (CRC)-specific mortality in total study population; Sensitivity analysis with additional adjustment for BMI, smoking status and CCI.

| Leukocyte composition | Categories | All-cause mortality | | CRC-specific mortality | |
|---|---|---|---|---|---|
| | | $n_{death}/n_{cases}$ | HR (95%CI) * | $n_{death}/n_{cases}$ | HR (95%CI) * |
| **CD4+T cell** | Quartile 1 | 315/536 | Ref. | 190/532 | Ref. |
| | Quartile 2 | 281/545 | **0.86 (0.72, 1.02)** | 144/538 | **0.77 (0.61, 0.98)** |
| | Quartile 3 | 259/550 | **0.76 (0.63, 0.92)** | 143/550 | **0.75 (0.59, 0.96)** |
| | Quartile 4 | 206/549 | **0.63 (0.51, 0.77)** | 113/546 | **0.60 (0.46, 0.79)** |
| | Per SD increase | 1061/2180 | **0.84 (0.78, 0.91)** | 590/2166 | **0.83 (0.74, 0.92)** |
| **CD8+T cell** | Quartile 1 | 300/540 | Ref. | 173/534 | Ref. |
| | Quartile 2 | 268/546 | 0.85 (0.71, 1.01) | 140/543 | 0.77 (0.60, 0.98) |
| | Quartile 3 | 254/548 | 0.86 (0.71, 1.03) | 148/545 | 0.90 (0.71, 1.14) |
| | Quartile 4 | 239/546 | 0.84 (0.69, 1.02) | 129/544 | 0.87 (0.68, 1.12) |
| | Per SD increase | 1061/2180 | 0.96 (0.90, 1.04) | 590/2166 | 1.00 (0.91, 1.10) |
| **B cell** | Quartile 1 | 311/545 | Ref. | 183/543 | Ref. |
| | Quartile 2 | 281/547 | 0.98 (0.82, 1.16) | 150/543 | 0.95 (0.76, 1.20) |
| | Quartile 3 | 238/542 | **0.69 (0.57, 0.84)** | 128/539 | **0.67 (0.52, 0.86)** |
| | Quartile 4 | 231/546 | **0.70 (0.58, 0.85)** | 129/541 | **0.70 (0.55, 0.91)** |
| | Per SD increase | 1061/2180 | 0.91 (0.82, 1.01) | 590/2166 | 0.90 (0.78, 1.03) |
| **NK cell** | Quartile 1 | 278/545 | Ref. | 165/543 | Ref. |
| | Quartile 2 | 277/545 | 1.05 (0.88, 1.25) | 162/540 | 1.10 (0.87, 1.38) |
| | Quartile 3 | 247/544 | 0.93 (0.77, 1.13) | 129/541 | 0.98 (0.76, 1.27) |
| | Quartile 4 | 259/546 | 0.92 (0.75, 1.12) | 134/542 | 1.01 (0.77, 1.32) |
| | Per SD increase | 1061/2180 | 0.94 (0.87, 1.01) | 590/2166 | 0.95 (0.86, 1.05) |
| **Monocyte** | Quartile 1 | 260/545 | Ref. | 150/542 | Ref. |
| | Quartile 2 | 249/548 | 0.88 (0.73, 1.07) | 142/545 | 0.94 (0.74, 1.20) |
| | Quartile 3 | 277/545 | 1.15 (0.95, 1.38) | 136/542 | 1.08 (0.84, 1.38) |
| | Quartile 4 | 275/542 | 1.01 (0.84, 1.23) | 162/537 | 1.08 (0.84, 1.38) |
| | Per SD increase | 1061/2180 | 1.03 (0.96, 1.10) | 590/2166 | 1.02 (0.94, 1.11) |
| **Neutrophil** | Quartile 1 | 231/548 | Ref. | 127/546 | Ref. |
| | Quartile 2 | 246/550 | 1.14 (0.94, 1.37) | 124/548 | 1.06 (0.82, 1.38) |
| | Quartile 3 | 278/545 | 1.16 (0.94, 1.42) | 163/540 | 1.09 (0.83, 1.43) |
| | Quartile 4 | 306/537 | **1.44 (1.17, 1.78)** | 176/532 | 1.31 (0.99, 1.74) |
| | Per SD increase | 1061/2180 | **1.14 (1.05, 1.23)** | 590/2166 | **1.14 (1.03, 1.26)** |

Abbreviation: BMI, body mass index; CCI, Charlson comorbidity index; CI, confidence interval; HR, hazard ratio. Numbers printed in bold: statistically significantly different from 1 ($P < 0.05$). * HR was adjusted for age, sex, tumor stage, tumor subsite, timing of blood sampling, batch, body mass index, smoking status and Charlson comorbidity index. Complete case analysis was applied when adjusting for these variables with missing values.

**Table S4.** Associations of leukocyte composition with all-cause and colorectal cancer (CRC)-specific mortality among patients whose blood sample was taken prior to surgery, more than 1 month after surgery but not receiving chemo- or radiotherapy or more than 5 months after the first chemo- or radiotherapy

| Leukocyte composition | Categories | All-cause mortality | | CRC-specific mortality | |
|---|---|---|---|---|---|
| | | $n_{death}/n_{cases}$ | HR (95%CI)* | $n_{death}/n_{cases}$ | HR (95%CI)* |
| **CD4+T cell** | Quartile 1 | 94/145 | Ref. | 53/142 | Ref. |
| | Quartile 2 | 123/249 | **0.66 (0.48, 0.90)** | 55/247 | **0.59 (0.39, 0.90)** |
| | Quartile 3 | 121/275 | **0.56 (0.41, 0.78)** | 54/275 | **0.53 (0.34, 0.81)** |
| | Quartile 4 | 130/342 | **0.49 (0.35, 0.68)** | 63/342 | **0.47 (0.30, 0.74)** |
| | Per SD increase | 468/1011 | **0.80 (0.71, 0.90)** | 225/1006 | **0.77 (0.66, 0.91)** |
| **CD8+T cell** | Quartile 1 | 89/149 | Ref. | 41/147 | Ref. |
| | Quartile 2 | 98/201 | 0.75 (0.53, 1.06) | 42/201 | 0.62 (0.37, 1.04) |
| | Quartile 3 | 132/292 | 0.85 (0.62, 1.16) | 71/289 | 1.05 (0.67, 1.64) |
| | Quartile 4 | 149/369 | **0.68 (0.50, 0.94)** | 71/369 | 0.78 (0.49, 1.23) |
| | Per SD increase | 468/1011 | 0.92 (0.83, 1.02) | 225/1006 | 1.00 (0.86, 1.16) |
| **B cell** | Quartile 1 | 117/188 | Ref. | 61/187 | Ref. |
| | Quartile 2 | 121/243 | 0.81 (0.61, 1.07) | 54/242 | 0.90 (0.60, 1.35) |
| | Quartile 3 | 113/271 | **0.54 (0.40, 0.73)** | 53/269 | **0.52 (0.34, 0.78)** |
| | Quartile 4 | 117/309 | **0.52 (0.38, 0.71)** | 57/308 | **0.53 (0.36, 0.79)** |
| | Per SD increase | 468/1011 | 0.78 (0.59, 1.04) | 225/1006 | 0.86 (0.56, 1.31) |
| **NK cell** | Quartile 1 | 68/130 | Ref. | 38/127 | Ref. |
| | Quartile 2 | 100/211 | 1.13 (0.79, 1.62) | 49/211 | 1.27 (0.75, 2.15) |
| | Quartile 3 | 112/275 | **0.67 (0.46, 0.99)** | 51/275 | 0.87 (0.50, 1.51) |
| | Quartile 4 | 188/395 | 0.85 (0.59, 1.22) | 87/393 | 1.17 (0.70, 1.96) |
| | Per SD increase | 468/1011 | 0.89 (0.80, 0.98) | 225/1006 | 0.95 (0.83, 1.08) |
| **Monocyte** | Quartile 1 | 91/208 | Ref. | 44/208 | Ref. |
| | Quartile 2 | 80/218 | 0.81 (0.58, 1.12) | 35/217 | 0.88 (0.53, 1.45) |
| | Quartile 3 | 144/291 | 1.24 (0.93, 1.64) | 64/290 | 1.21 (0.79, 1.86) |
| | Quartile 4 | 153/294 | 1.18 (0.87, 1.60) | 82/291 | 1.34 (0.85, 2.11) |
| | Per SD increase | 468/1011 | 1.07 (0.97, 1.18) | 225/1006 | 1.07 (0.93, 1.21) |
| **Neutrophil** | Quartile 1 | 152/382 | Ref. | 75/382 | Ref. |
| | Quartile 2 | 142/318 | **1.31 (1.04, 1.66)** | 60/317 | 1.16 (0.81, 1.67) |
| | Quartile 3 | 105/197 | **1.39 (1.04, 1.85)** | 55/196 | 1.36 (0.91, 2.02) |
| | Quartile 4 | 69/114 | **1.91 (1.30, 1.85)** | 35/111 | 1.45 (0.85, 2.49) |
| | Per SD increase | 468/1011 | **1.26 (1.11, 1.43)** | 225/1006 | 1.17 (0.97, 1.41) |

Abbreviation: CI, confidence interval; HR, hazard ratio. Numbers printed in bold: statistically significantly different from 1 ($P < 0.05$). * HR was adjusted for age, sex, tumor stage, tumor subsite, timing of blood sampling, and batch. Complete case analysis was applied when adjusting for these variables with missing values.

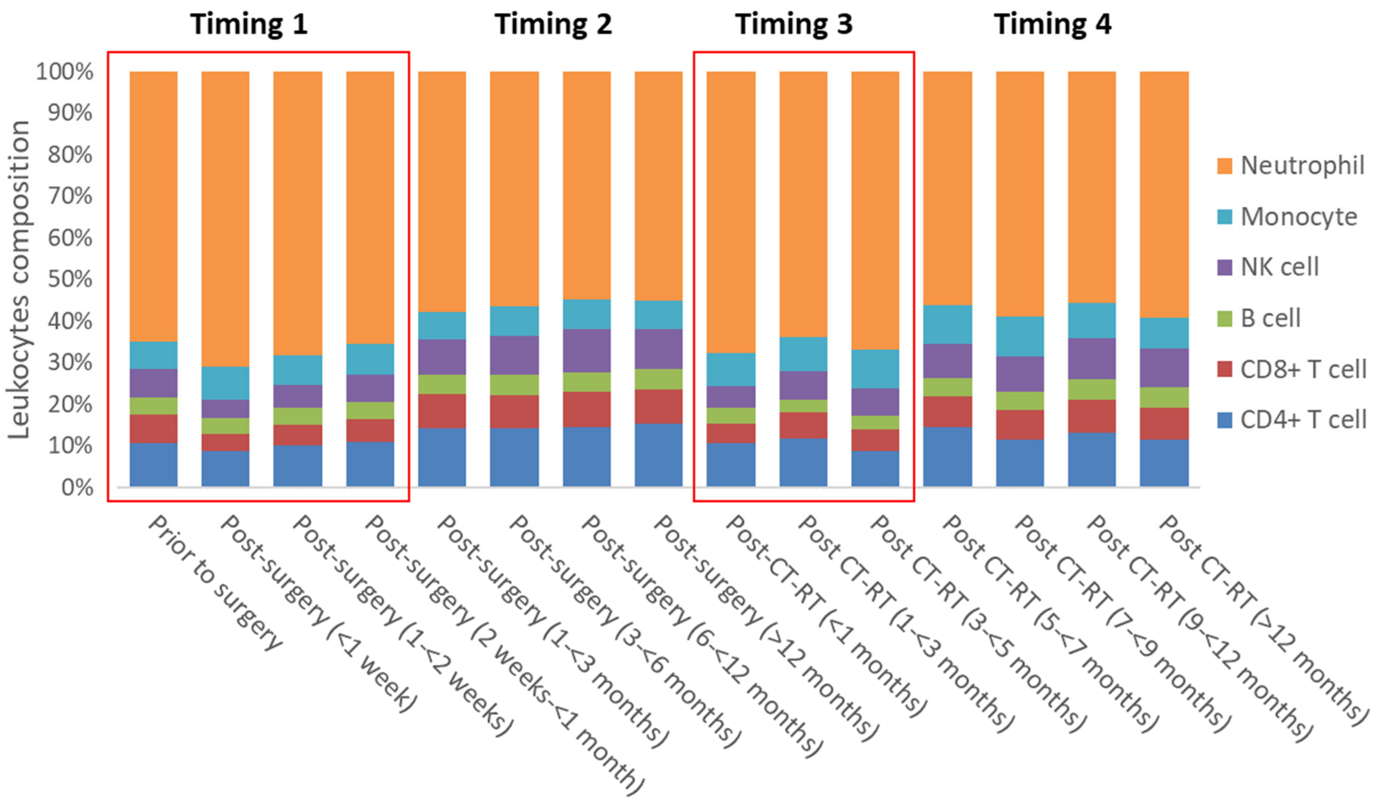


**Figure S1.** Leukocytes proportion according to the time of blood collection relative to treatments. Note: (a) CT-RT: chemotherapy or radiotherapy; The "post-surgery" categories include patients whose blood were collected after receiving surgery but before or not receiving chemotherapy or radiotherapy; The "post CT-RT" categories include patients whose blood samples were collected after receiving chemotherapy or radiotherapy. (b) The timing of blood sampling were grouped into four categories (1) prior to surgery and within 1 month after surgery (*n* = 1,006); (2) more than 1 month after surgery, but not receiving chemo- or radiotherapy (*n* = 569); (3) within 5 months after the first chemo- or radiotherapy (*n* = 278); (4) more than 5 months after the first chemo- or radiotherapy (*n* = 353).

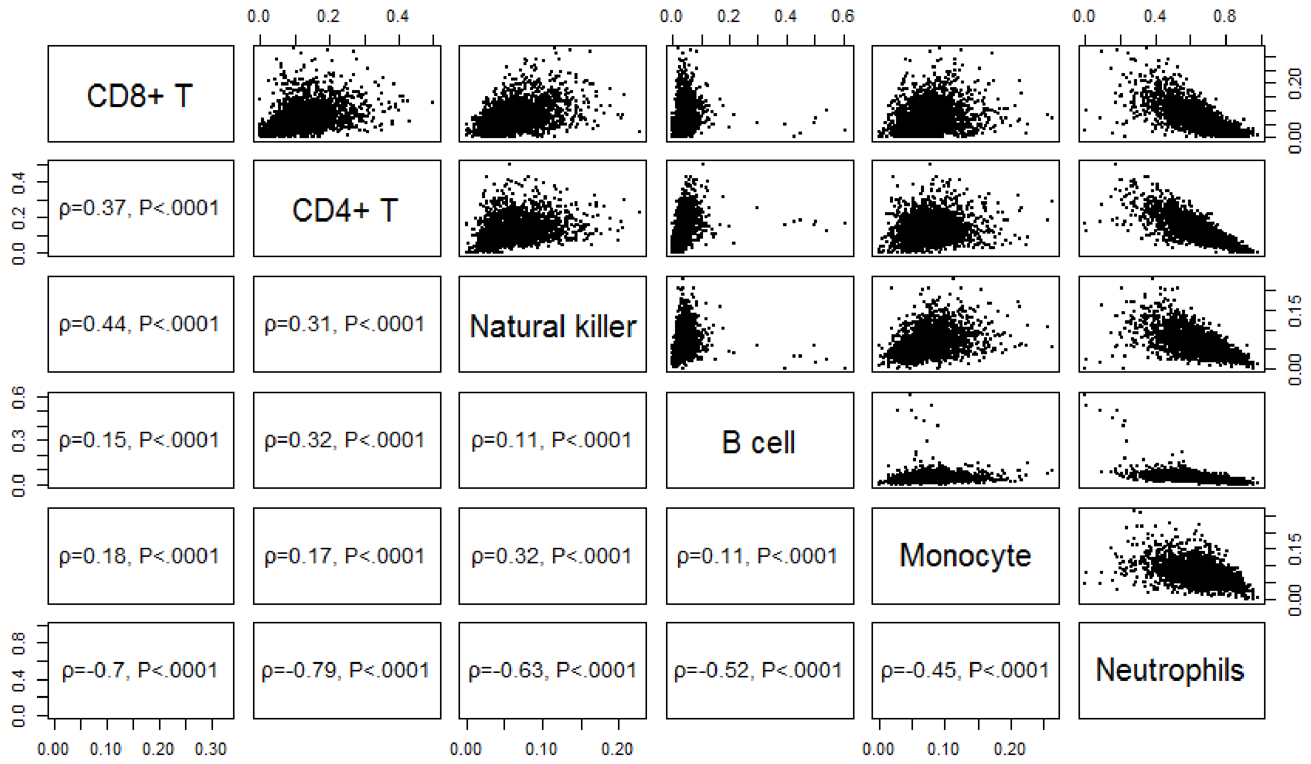


**Figure S2**. Correlations of leukocyte subtype proportions. ρ: Pearson correlation coefficient.

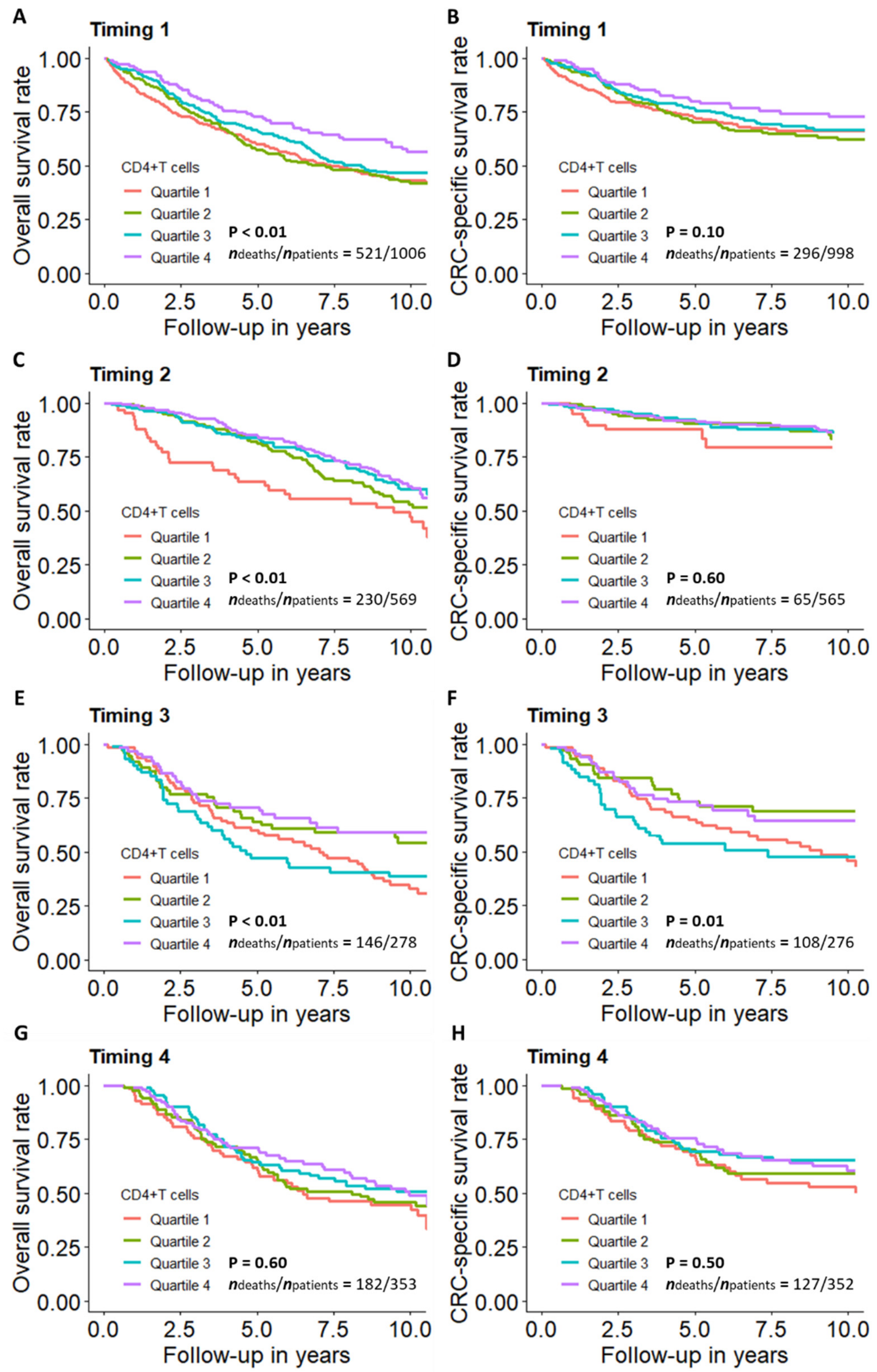


**Figure S3.** Survival curves for CRC patients across quartiles of CD4+ T cells proportion by the timing of blood sampling. Note: (A) overall and (B) CRC-specific survival curve among patients whose blood was sampled prior to surgery and within 1 month after surgery; (C) overall and (D) CRC-specific survival curve among patients whose blood was sampled more than 1 month after surgery, but not receiving chemo- or radiotherapy; (E) overall and (F) CRC-specific survival curve among patients whose blood was sampled within 5 months after chemo- or radiotherapy; (G) overall and (H) CRC-specific survival curve among patients whose blood was sampled more than 5 months after chemo- or radiotherapy. HR was adjusted for age, sex, tumor stage, subsite and batch. Quartile 1: lowest quartile; quartile 4: highest quartile.

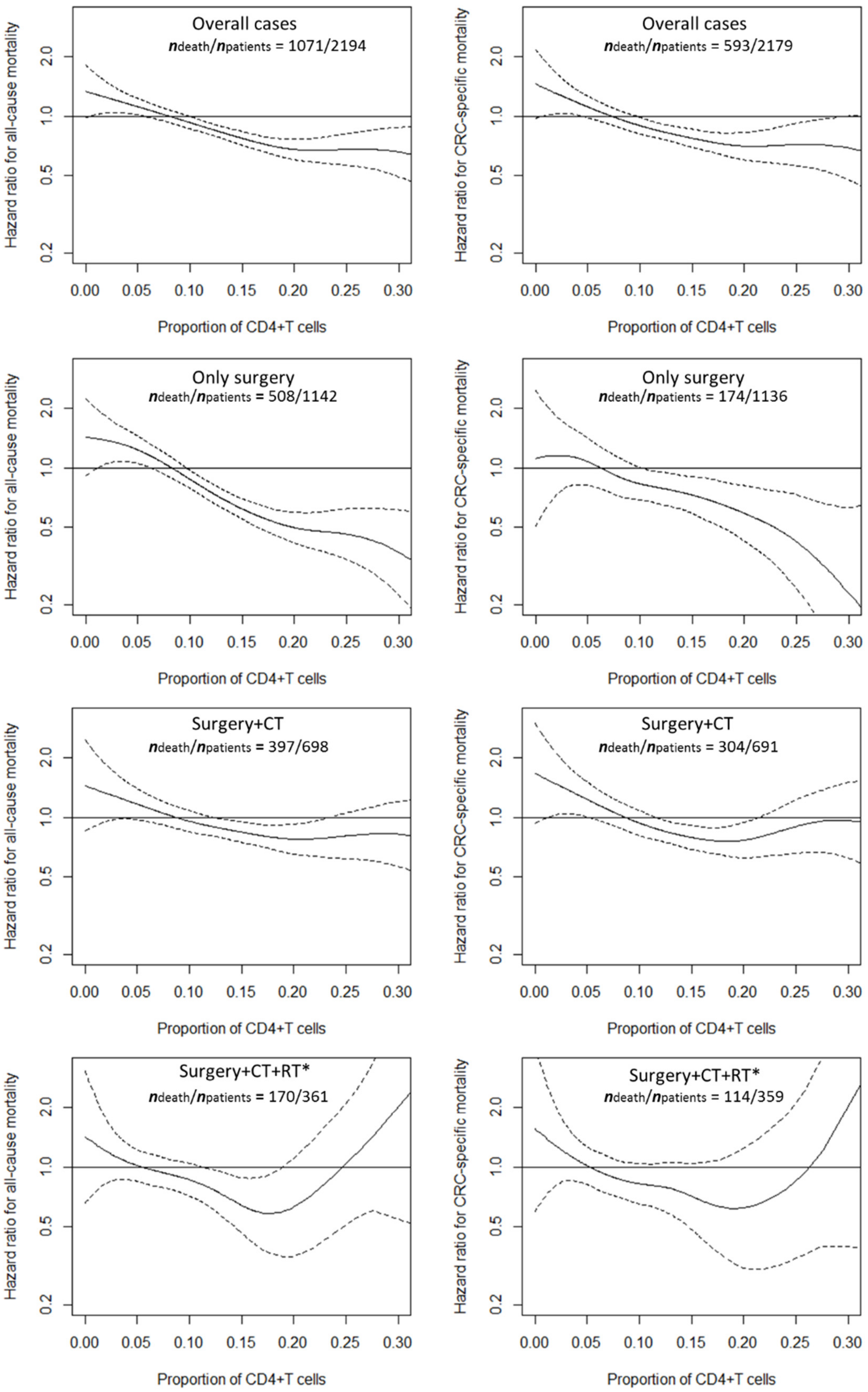


**Figure S4**. Adjusted restricted cubic splines for CRC patients across quartiles of CD4+ T cells proportion by therapies. Note: Hazard ratios were adjusted for age, sex, tumor stage, tumor subsite, timing of blood sampling and batch; 25th percentile of CD4+ T cells is set as the reference; In the group “surgery+CT+RT*”, 42 patients only received surgery and radiotherapy; Abbreviations: CT, chemotherapy; RT, radiotherapy.
